# Is It Time for a Change? A Cost-Effectiveness Analysis Comparing a Multidisciplinary Integrated Care Model for Residential Homes to Usual Care

**DOI:** 10.1371/journal.pone.0037444

**Published:** 2012-05-24

**Authors:** Janet L. MacNeil Vroomen, Marijke Boorsma, Judith E. Bosmans, Dinnus H. M. Frijters, Giel Nijpels, Hein P. J. van Hout

**Affiliations:** 1 Department of General Practice, VU University Medical Center, EMGO Institute for Health and Care Research, Amsterdam, The Netherlands; 2 Section Geriatrics, Department of Internal Medicine, Amsterdam Medical Center, Amsterdam, The Netherlands; 3 Department of Health Sciences, Faculty of Earth and Life Sciences, VU University Amsterdam, EMGO Institute for Health and Care Research, Amsterdam, The Netherlands; 4 Department of Nursing Home Practice, VU University Medical Center, EMGO Institute for Health and Care Research, Amsterdam, The Netherlands; 5 VU University Medical Center, EMGO Institute for Health and Care Research, Amsterdam, The Netherlands; RAND Corporation, United States of America

## Abstract

**Objective:**

The objective of this study was to evaluate the cost-effectiveness of a Multidisciplinary Integrated Care (MIC) model compared to Usual Care (UC) in Dutch residential homes.

**Methods:**

The economic evaluation was conducted from a societal perspective alongside a 6 month, clustered, randomized controlled trial involving 10 Dutch residential homes. Outcome measures included a quality of care weighted sum score, functional health (COOP WONCA) and Quality Adjusted Life-Years (QALY). Missing cost and effect data were imputed using multiple imputation. Bootstrapping was used to analyze differences in costs and cost-effectiveness.

**Results:**

The quality of care sum score in MIC was significantly higher than in UC. The other primary outcomes showed no significant differences between the MIC and UC. The costs of providing MIC were approximately €225 per patient. Total costs were €2,061 in the MIC group and €1,656 for the UC group (mean difference €405, 95% −13; 826). The probability that the MIC was cost-effective in comparison with UC was 0.95 or more for ceiling ratios larger than €129 regarding patient related quality of care. Cost-effectiveness planes showed that the MIC model was not cost-effective compared to UC for the other outcomes.

**Interpretation:**

Clinical effect differences between the groups were small but quality of care was significantly improved in the MIC group. Short term costs for MIC were higher. Future studies should focus on longer term economic and clinical effects.

**Trial Registration:**

Controlled-Trials.com ISRCTN11076857

## Introduction

In nearly every country around the world, the proportion of people aged over 60 years is growing faster than any other age group [1]. Long-term care costs are largely affected by this increase because long-term care expenses tend to increase markedly with old age [2]. As the aging population intensifies its demand and uptake of healthcare services, the contextual landscape is one of a decreasing labor market, higher demands for quality of care voiced by baby boomers, and uncertainty of incomes of older people [3], [4].

Approximately 10% of all Dutch elderly over the age of 75 live in elderly housing [5], [6]. Of this population, over 70% require professional assistance with activities of daily living, nursing care and housekeeping [5], [6]. There are approximately 100 residents per residential home [3]. When senior citizens enter into a residential home, they keep their general practitioner if possible. There is a trend to keep the elderly in their own homes for as long as possible to maximize their level of independence as well as it can be less expensive from a governmental perspective [7], [8]. As a consequence, the residential home population resembles nursing home populations more and more [9], [10], [11], [12]. Residential homes were not designed to address these populations and primary care physicians are challenged by these complex patients [9], [13], [14]. Most care organizations want to innovate and improve their quality of care but lack expertise or financial resources [9], [13], [15]. The Multidisciplinary Integrated Care (MIC) model is inspired by the chronic care model [16], [17] and is a multidisciplinary approach that may improve quality of care [18]. The objective of this study was to determine the cost-effectiveness of the MIC model compared to usual care (UC) in a sample of 10 residential homes in the Netherlands. In an earlier paper, it was found that the MIC model resulted in significantly higher quality of care [18].

## Methods

### Design and setting

A clustered, randomized controlled trial with 6 month follow-up was conducted in 10 Dutch residential homes [9]. Residential homes were randomized to either the intervention or control group resulting in each arm of the trial including 5 residential homes. A detailed description of the design was published elsewhere [9], [18]. The protocol for this trial and supporting CONSORT checklist are available as supporting information; see Protocol S1 and Checklist S1. Randomization was carried out on at the level of care facilities after matching for percentage of cognitively impaired residents, based on the assumption that a high percentage of cognitive residents would affect care-related needs and services. In the matching procedure, the two facilities with the highest percentage of cognitively impaired residents were matched, and so on. Randomization was carried out using the first column from Pocock's random numbers table. The average number of residents in each facility was 46, and staff included nurse assistants and a house manager.

### Ethics statement

The ethical committee of the VU Medical Center approved the study.

### Resident selection

Patients were recruited from December 2006 until December 2007. All residents within the 10 residential homes were invited to participate in the clinical trial. A patient was excluded from the study if he/she was viewed by the staff or primary care physician as too terminally ill to complete the study [9]. All residents were listed at a general practitioner who was responsible for their medical care. Participating residents in each facility were visited by trained, blinded interviewers at baseline and at six months to assess other outcomes.

If the resident was unable to understand the questions, a close family member was identified by staff and asked to act as a proxy. The interview consisted of a computerized assessment of functional health, activities of daily living, depression, cognition, satisfaction with care, and use of medications. All participants or their representative signed informed consent.

### The UC model

A residential home is a retirement home for seniors who can no longer live independently [19]. Residential homes typically offer general care such as; domestic help, leisure activities and meals for all occupants or a large portion of the occupants [19]. Ad hoc nursing care for individual occupants is also possible. No new interventions were introduced into this arm of the study. Care providers were instructed to continue the care to the patients that they would normally provide.

### The MIC

The intervention of the MIC model consists of three steps [9]. Firstly, a quarterly in-home systematic and computerized multidimensional assessment of all residents by trained nurse-assistants systematically reviewed the functional health status and care needs using the InterRAI-LTCF which is a comprehensive, standardized instrument for evaluating the needs, strengths, and preferences of those in chronic care and nursing home institutional settings [20]. The InterRAI-LTCF assessment form incorporates domains such as; function, mental and physical health, social support, medication and service use [20]. The problem areas identified become the foundation for the individual care plan [20]. Secondly, the outcomes of the assessment were discussed in a multidisciplinary meeting in the homes with the primary care physician, nursing home physician, nurse, psychotherapist and other involved disciplines. Lastly, a multidisciplinary consultation was offered to the frailest residents with complex health care problems which were identified by the level of expected resource utilization [9], [21].

### Clinical outcomes measures

The primary outcome was the sum score of the 32 risk-adjusted quality-of-care indicators [18]. The quality-of care indicators were based on observations recorded in the Long-term Care Facility assessment form [22]. The itemized observations needed to calculate these indicators were rated by independent trained interviewers. Inter-rater reliability of the quality-of-care indicators between interviewers and nurse-assistants in the intervention facilities was satisfactory (mean intra-cluster correlation single measure 0.74). The sum score of the quality-of-care indicators was determined by the number of indicators that were present per resident divided by the number of applicable indicators per resident. An example of a quality indicator is the presence of a feeding tube. Lower sum scores indicate higher quality of care.

Functional health, an important aspect of quality of life, was measured by COOP WONCA charts [23]. The COOPWonca chart consists of six dimensions: physical fitness, feelings, daily activities, social activities, change in health and overall health. These dimensions combined form a total COOPWonca score. Higher scores are indicative of better functional health.

The 12- Item Short Form health survey (SF12) was used to measure general quality of life. Based on The SF12 data, Quality Adjusted Life Years (QALY) were calculated using utility scores estimated by the SF6D tariff [24]. Transitions between health states were linearly interpolated.

### Cost outcome measures

Cost data were collected at baseline and six months from a societal perspective. Health care utilization data was collected by patient or proxy interview and medical records at baseline and at six months [9]. Table 1 lists the cost categories and prices used in the economic evaluation. All prices were adjusted for the year 2007 using consumer price index figures [25]. Costs of medications were valued using prices from the Royal Dutch Society for Pharmacy [26]. We calculated informal care hours, primary and secondary care consumption, medication use and costs associated with the intervention. Normally productivity costs are included but this is an admitted population therefore the costs were not relevant.

**Table 1:** Costs – in the economic evaluation using consumer price index figures (in Euros) [25].

Cost category	€ (2007)
Primary care costs	
General practitioner	
-Visit to GP (per visit)	21.36
-Visit from GP (per visit)	42.73
-Contact by telephone	10.66
Physical therapy	
-Physiotherapy (per visit)	22.40
-Ergotherapy (per visit)	53.03
Psychosocial therapy	
-Psychologist (per visit)	81.02
-Psychiatrist (per visit)	80.38
-Social psychiatric nurse (per visit)	80.38
Secondary care costs	
Medical specialist	
-Geriatrician (per visit)	177.69
-Other specialists (per visit)	59.23
Admission to hospital	
-Day care (per day)*	242.15
-Overnight stay (per day)*	353.35
Informal care (per hour)	8.78
MIC costs	€
-Organizational costs	2,510
-Training of staff	6,824
-Performing interRAI	1,999
-Meeting costs	1,780
Total costs	13,113
Cost per patient	225

* Price including costs medical specialist, nurses, medication, housing costs, medical equipment.

A cost price for MIC was calculated using a top down approach. Total costs included: organizational costs, training costs, InterRAI costs and multidisciplinary meeting costs (see Table 1). Costs were calculated on an annual basis and then proportioned for the six month trial. Total costs of the intervention were divided by the total number of residents living in the intervention residential homes. Multidisciplinary meetings are part of usual care by law. However, in daily practice, not all homes hold these meetings on a regular basis. We also calculated costs for the meetings held in the usual care home. In a sensitivity analysis, only the license costs of the InterRAI and the InterRAI subscription costs per patient were included.

### Statistical analysis

Data was analyzed according to the intention to treat principle. However, patients who did not provide baseline data or died during the study were excluded from the analyses. The multiple imputation function in SPSS-18 was used to predict missing values for cost and effect data. This function created five imputed data sets that were pooled together using Rubin's rules [27]. Individual cost components were imputed at a patient level instead of overall total cost per patient to minimize unnecessary deletion of information.

As patient-level cost data have a highly skewed distribution, bootstrapping was performed with 5000 replications to estimate Approximate Bootstrap Confidence (ABC) intervals around cost differences [28], [29]. Incremental cost-effectiveness ratios (ICERs) were calculated by dividing the difference in total costs between MIC and UC by the difference in clinical effects. Non-parametric bootstrapping was also used to estimate the uncertainty surrounding the ICERs (5000 replications). The bootstrapped cost-effect pairs were plotted on a cost-effectiveness plane (CE plane) [30] and used to estimate cost-effectiveness acceptability curves (CEA curves). CEA curves illustrate the probability that the intervention is cost-effective in comparison with the control treatment for a range of ceiling ratios. The ceiling ratio is defined as the societal willingness to pay in order to gain one unit of effect [31].

Three sensitivity analyses were performed. One included only the complete cases and the second one included only the licensing and subscription costs of the interRAI as described above. In the third sensitivity analysis, people who provided no baseline data or died were included in the analysis. Missing cost and effect data were imputed based multiple imputation of available baseline clinical and cost data.

## Results

From December 2006 until December 2007, a total of 462 residents were requested to participate in the trial. There were 340 patients randomized. At baseline, 340 people were included (201 intervention patients and 139 control patients). There were no significant differences in patient characteristics between the two groups at baseline (Table 2). There were no baseline data for 5 patients (2 intervention and 3 control patients). A total of 34 people died (16 (12%) control and 18 (9%) intervention patients) before the six month follow up. Thus, all main analyses were based on imputed data including 181 intervention and 120 control residents. Complete clinical outcome data was available for 137 patients (68%) in the intervention group and 70 (50%) patients in the control group. Selectively missing data was found as the participants that dropped out were approximately two years older (95% CI 0.42; 3.66) and had better activities of daily living score as measured by the Groningen Activity Restriction Scale (GARS) compared to completers (mean difference −3.4, 95% CI −6.7; −0.1).

**Table 2:** Mean (SD) baseline characteristics of intervention and control groups.

	Intervention (N = 201)	Control (N = 136)
Mean age	86 (6.2)	85 (8.0)
Female (%)	76	74
Education		
-Primary school or less	112 (56)	79 (58)
-Lower Technical vocational training	45 (22)	26 (19)
-Average and higher vocational training	34 (17)	30 (22)
-Missing	10 (5)	1 (1)
Marital status, n (%)		
-Married	42 (21)	27 (20)
-Widowed	130 (65)	93 (68)
-Single	19 (9)	15 (11)
-Missing	10 (5)	1 (1)
Physical Component Scale of the SF 12	34 (8.3)	33 (7.2)
Mental Component Scale of the SF 12	53 (9.3)	51(11.1)
Baseline utility SF-6D	0.64 (0.1)	0.64 (0.1)
COOP WONCA	18 (3.7)	18 (4.1)

### Clinical effectiveness

Quality of care was significantly higher in the intervention group than the control group (mean difference −6.5, 95% CI −9.5; −3.5). However, there was no statistically significant difference in effect for either of the other outcome measures (Table 3). Mean QALY scores for both groups were approximately 0.3 (n = 181 for intervention group and n = 120 for the usual care group) indicating that there was no difference in quality of life over the six month study.

**Table 3:** Differences in clinical outcomes at 6 months.

Outcome measure	MIC (n = 181)	UC (n = 120)	Difference (95% CI)
Primary outcomes			
Quality Indicator Score*	11.12 (1.1)	17.63 (1.0)	−6.5 (−9.5; −3.5)
COOP WONCA	0.85 (0.3)	0.65 (0.6)	0.2 (−1.1; 1.5)
QALY	0.31 (0.003)	0.32 (0.004)	0.00 (−0.01; 0.01)

* Lower scores indicate better quality of care.

### Costs

Costs of the intervention amounted to €225. There was a trend that total costs were higher in the intervention group compared to UC by €404 (95% CI −13; 826, Table 4). Direct healthcare costs were the largest contributor to total costs in both groups. The highest cost driver within direct healthcare costs for both groups was secondary care costs such as hospital admission (Table 4).

**Table 4:** Mean (SD) and cost differences € (95% CI) during follow-up at 6 months.

Cost category	Intervention (n = 181)	Control (n = 120)	Difference
Direct costs			
-Direct healthcare costs	1,469 (158)	1,351 (161)	117 (−292; 529)
Primary care costs	299 (37)	389 (74)	−88 (−277; 48)
Secondary care costs	745 (143)	533 (135)	215 (−146; 579)
Medications	419 (40)	429 (31)	−8 (−84; 114)
-Informal care costs	367 (47)	282 (32)	77 (−10; 204)
-Implementation costs*	225	23	202
Total costs	2,061 (163)	1,656 (163)	405 (−13; 826)

* Implementation costs consist of the MIC costs in the intervention group and of the costs of the multi-disciplinary meetings in the control group.

### Cost-effectiveness analysis

#### Quality Indicators

**Figure 1:**
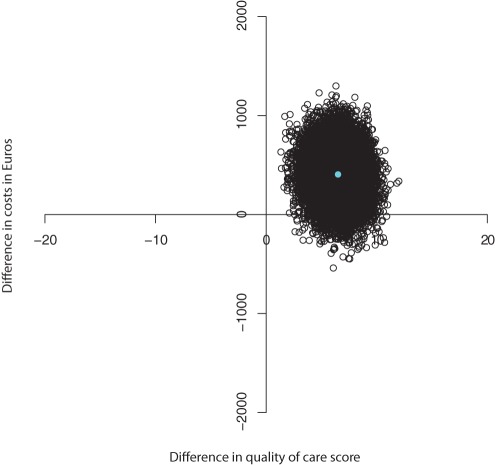
Cost-effectiveness plane for the difference in quality of care sum score at 6 months (in Euros).

**Figure 2:**
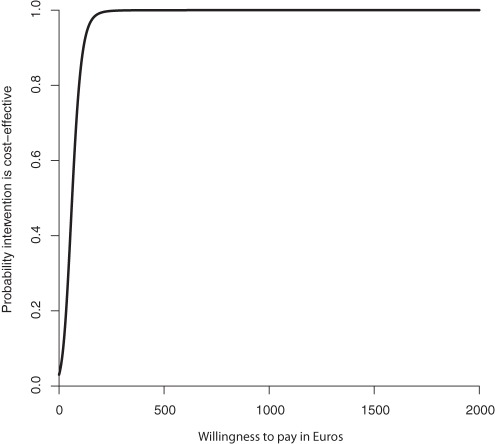
Cost- effectiveness acceptability curve for the quality of care sum score at 6 months (in Euros).

The sum score of quality of care resulted in a negative ICER of 62, indicating that for every one point improvement on the sum score, the MIC model costs €62 compared to UC. Figures 1 and 2 show the CE plane and CEA curve. The majority of the cost- effectiveness pairs (97%) were in the northeast quadrant suggesting that the intervention is more significantly more effective and more costly than UC. The CEA curve showed that with a 0.95 probability that the MIC was cost-effective compared to UC the societal willingness to pay should be approximately €129 or more per point of improvement on the quality of care scale.

#### Coop WONCA

The ICER for the COOP WONCA was 2,056 meaning that 1 point improvement in COOP WONCA score costs €2,056 for MIC versus UC. The majority (97%) of the cost-effect pairs fell in the Northern quadrants of the CEA plane indicating that total costs in the MIC are higher compared to UC while there is a statistically non-significant difference in effects. The CEA curve showed that the maximum probability that the MIC was cost-effective compared to UC was 0.6. However, to reach this probability the societal willingness to pay should be approximately €5,000 per one point improvement.

#### QALY

**Figure 3:**
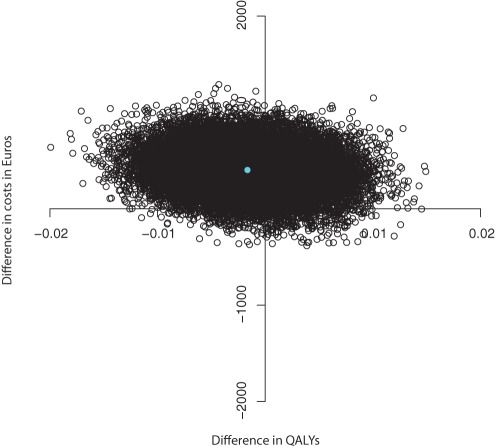
Cost-effectiveness plane for the differences in QALY scores at 6 months (in Euros).

**Figure 4:**
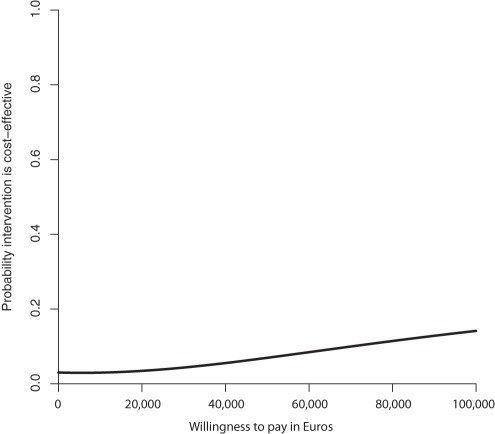
QALY Cost-effectiveness acceptability curve at 6 months (in Euros).

The ICER for QALY scores was −248,308 indicating the MIC had higher costs and negative effects compared to UC. Figures 3 and 4 show the CE plane and CEA curve. Most (63%) bootstrapped cost effect pairs were contained in the Northwest quadrant meaning that the MIC was less effective and more costly than UC. The CEA curve presented in Figure 4 shows that the maximum probability that MIC is cost-effective in comparison with usual care was 0.14 regardless of the willingness to pay.

### Sensitivity analysis

The results of the clinical outcomes in the complete case analysis were consistent with those of the imputation analysis. Total costs were higher in the intervention group than in the control group but not statistically significantly which is consistent with the imputed analysis. Although the conclusion for the cost effectiveness analysis was the same for both the imputed and complete case analysis the numbers varied (data not shown).

The second sensitivity analysis including only licensing and subscription costs for interRAI showed that total costs were not significantly different between the intervention and the control group. Conclusions on the cost-effectiveness of MIC in comparison with usual care did not change in this analysis.

The third sensitivity analysis included people who died between baseline and six months follow-up in addition to the participants who missed the baseline measurement. We found that there was no significant difference in costs between the intervention and the control.

## Discussion

### Summary

An economic evaluation was performed to determine whether the MIC was cost-effective compared to UC. General scales of functional health did not significantly differ between the groups at six month although quality of care was significantly higher in the MIC group. There was a trend that total costs were higher in the MIC than UC. For functional health and QALYs we concluded that the MIC was not cost-effective compared to UC. Whether MIC is considered cost-effective in comparison with UC for quality of care depends on the amount of money decision makers are willing to additionally spend on care for this group of elderly nursing home residents. Conclusions were similar in the complete case analysis.

### Explanation of the findings

This raises the question why was quality of care higher in the intervention homes compared to the control homes? It is possible the quality indicators in the control homes did not improve to the same extent as in the intervention homes because intervention participants were receiving increased attention from the residential home staff as well as increased referrals to secondary care. The increase in secondary care may have induced the need for the informal caregiver to attend and help transport patients to the secondary care appointments which may explain the increased informal care costs. If there was unmet care, then the use of the interRAI and the multidisciplinary meetings addressed this gap in care. However, a trade-off needs to be made whether the additional effects are worth the additional costs.

### Existing literature comparison

Previous studies suggest interRAI has positive effects on health outcomes in nursing facilities as well as in residential homes [32], [33]. However, there were criticisms on the study designs and the conclusions drawn indicating a need for better designed trials [34]. A four month trial from New Zealand estimated health care services utilized and the cost of implementing the minimum data set home care assessment compared with UC [35]. They found that the interRAI was significantly more costly in prescribed and delivered services compared to UC but the author believed that the cost differences may be due to a genuine need of services for this population [35]. We think that our trial is an important addition to the knowledge base on the effect of the interRAI in clinical care.

### Limitations

The six month follow-up may not have been enough to capture all potential costs and effects. The duration of the trial was relatively short because of a high risk for drop out owing to the extreme vulnerability of residents and because the umbrella care organization intended to implement the care model in the control facilities as well. Patients in a residential home have a heterogeneous mix of chronic conditions that naturally erode health over time which makes it difficult to know if an intervention of this sort would be able to override the downward trend of health states associated with chronic conditions in such a short time span. The primary outcome variables may not have been sensitive enough to pick up differences within such a limited time interval. Another limitation was the considerable amount of missing data. In this study, non-completers tended to be older and had better activities of daily living scores. As the intervention really targeted only the frailest it could be that they did not feel like they were benefitting enough from the study intervention. In situations where there are missing costs, multiple imputation is recommended which was also performed in this study.

### Conclusion

This study showed benefit on quality of care, against a modest cost increase. Longer term follow up of costs and effects is needed to further substantiate the findings. Future research should consider the reasons why it did not translate over to the other clinical outcome variables. Its pragmatic study design resembles clinical practice to a high degree which increases the relevance of the study results.

Future research should consider the reasons why these patients in the Multidisciplinary Integrated Care group had higher quality of care indicators and why it did not translate over to the other clinical outcome variables. Moreover, ways to decrease MIC implementation costs could be beneficial for future cost-effectiveness analyses.

## Supporting Information

Checklist S1 **CONSORT checklist.**
(DOC)

Protocol S1 **Trial Protocol.**
(PDF)
