# Supplementary material for: Is It Time for a Change? A Cost-Effectiveness Analysis Comparing a Multidisciplinary Integrated Care Model for Residential Homes to Usual Care
Source: PLoS One. 2012 May 24;7(5):e37444. doi: 10.1371/journal.pone.0037444 (PMC3360056; doi:10.1371/journal.pone.0037444)
Supplement: Checklist S1 — CONSORT checklist. (DOC) [file pone.0037444.s001.doc]

Table 1: CONSORT checklist

Allocation

Analysis

Follow-Up

Enrollment

Assessed for eligibility (n=462)

)

Excluded (n=122)

♦  Not meeting inclusion criteria (n=52)

| ♦ Died 35 |
| --- |
| ♦ Admitted to hospital 2 |
| ♦ Admitted to nursing home 15 |

♦  Declined to participate (n=42 )

♦  Other reasons (n=28 )

| Main Cost-effective analysis: (n=181 ) |
| --- |
| Sensitivity analysis: complete case (n=137)  Sensitivity analysis: including dead and missing baseline  people (n=201) |

Lost to follow-up (n=20)

| ♦Died | 18 |
| --- | --- |
| ♦People who missed baseline | 2 |
|  |  |
|  |  |

Discontinued intervention (refusal) (n= 18)

Allocated to intervention (n=201)

♦ Received allocated intervention (n=201)

♦ Did not receive allocated intervention (n=0)

Lost to follow-up (n=19)

| ♦Died | 16 |
| --- | --- |
| ♦People who missed baseline | 3 |
|  |  |
|  |  |

Allocated to intervention (n=139)

♦ Received allocated intervention (n=139)

♦ Did not receive allocated intervention (n=0)

| Main Cost-effective analysis: (n=120 ) |
| --- |
| Sensitivity analysis: complete case (n=70)  Sensitivity analysis: including dead and missing  baseline people (n=139) |

Randomized (n=340)
