# Supplementary material for: Is It Time for a Change? A Cost-Effectiveness Analysis Comparing a Multidisciplinary Integrated Care Model for Residential Homes to Usual Care
Source: PLoS One. 2012 May 24;7(5):e37444. doi: 10.1371/journal.pone.0037444 (PMC3360056; doi:10.1371/journal.pone.0037444)
Supplement: Protocol S1 — Trial Protocol. (PDF) [file pone.0037444.s002.pdf]

(METC*)* version 5, HvH 18 April 2006

# NL:

# Preventieve effecten van ketenzorg op bewoners in verzorgingshuizen, een gerandomiseerd gecontroleerd klinisch experiment

# UK:

# Preventive effects of Transmural Integrated Care (´ketenzorg´) on residents in homes for the elderly, a randomised controlled clinical trial

ZONmw DO, Sub programme: early evaluation of medical innovation



| Naam | Organisatie | Functie | Rol |
|---|---|---|---|
| HPJ van Hout | Vrije Universiteit Medisch Centrum | Senior onderzoeker | Projectleider en penvoerder |
| G Nijpels | Vrije Universiteit Medisch Centrum | Huisarts, senior onderzoeker | Projectleider |
| M Ribbe | Vrije Universiteit Medisch Centrum | Hoofd Afdeling Verpleeghuisgeneeskunde | Commissielid |
| ME de Bruijne | Vrije Universiteit Medisch Centrum | HTA senior | Adviseur |
| M van Tulder | Vrije Universiteit Medisch Centrum | HTA senior | Adviseur |
| WAB Stalman | Vrije Universiteit Medisch Centrum | Huisarts, hoofd afdeling Huisartsgeneeskunde | Bestuurlijk verantwoordelijke |
| M Boorsma | West-Friese Zorggroep De Omring | Verpleeghuisarts | Uitvoerend onderzoeker klinisch deel |
| D Frijters | Vrije Universiteit Medisch Centrum & Prismant | Senior Verpleeghuisgeneeskunde & consultant Prismant | Commissielid |



**Summary (max 50 lines)**


*-Background:*
Persons in homes of the elderly suffer frequently from (multiple) chronic diseases. Transmural Integrated Care (TIC) is strongly recommended for (chronic) patients. Little is known about the cost-effectiveness of TIC in this setting.
*-Objectives:*
(1) To determine the cost-effectiveness of TIC (ketenzorg) for residents in homes for the elderly.
(2) To identify residents who benefit most from the intervention.
*-Methods:*
A randomised two-group controlled clinical trial, among residents of elderly homes of 5 intervention homes and 5 usual care homes in West-Friesland that comprise over 500 residents. Houses are randomised after matching in pairs on percentage psychogeriatric patients.
*-Population:*
Exclusion criteria are terminally ill, waiting for nursing home placement, cognitive impairment (MIS<2).
*-Intervention:* TIC is on a process level operationalised in three sequential elements. Firstly, an in home multidimensional assessment is carried out by trained staff (nurse) of the patients functional health and care needs with the Resident Assessment Instrument (RAI). Computerisation of the RAI enables immediate identification of problem areas and guides individualised care plans. Secondly, the assessment outcomes are discussed in a multidisciplinary consultation (MC) with the nurse, GP, nursing home phyisician, and psychologist. The MC presents individualised care plans to manage or treat modifiable disabilities and risk factors. Thirdly, consultation by a nursing home physician and psychologist is offered to the frailest residents at risk for nursing home admission (according to the RAI).
*-Outcome Measures*
Primary: Quality of Care (Zimmerman criteria); Quality Adjusted Life Years (Euroqol); Functional health (COOP-WONCA & SF12)

Secondary: disability (GARS); Patients´ care satisfaction (brief QUOTE); Mood (PRiME-MD); hospital days; time to nursing home; and to mortality.
Carers of incompetent residents are interviewed for proxy information on outcomes.
*. Power/data analysis:*
We strive to include 166 residents which is powered to detect a difference in the primary outcomes with an effect size of 0.5. (beta =0.2; alfa=0.05; anticipating intracluster inflation of 10% and 15% loss to follow up).
*. Economic evaluation:*
The costs are measured from societal perspective. Both direct (e.g. care consumption) and indirect (e.g. care time by residential workers) are measured. Cost-effectiveness analyses will be done for the primary outcomes.
*. Time schedule:*
Total 2 years: 12 months inclusion, 6 months follow up, 6 months analyses and reporting.

**Samenvatting**
*. Achtergrond:*
Verzorgingshuisbewoners lijden in hoge mate aan (multipele) chronische ziekten. Ketenzorg wordt sterk aanbevolen om de kwaliteit van zorg te verbeteren voor chronische patienten. Er is echter zeer weinig bewijs voor de kosten-effectiviteit van ketenzorg in deze setting.
*. Doel / vraagstelling:*
1) Wat is de kosten-effectiviteit van ketenzorg voor bewoners van verzorgingshuizen?
2) Welke bewoners in verzorgingshuizen hebben het meeste baat bij ketenzorg?
*. Studie-opzet:*
Een gerandomiseerd gecontroleerd experiment in 5 interventie en 5 verzorgingshuizen met gebruikelijk zorg in West-Friesland. Randomisatie vindt plaats op huis na matching in tweetallen op percentage psychogeriatrische patiënten.
*. Studiepopulatie:*
De verzorgingshuizen hebben gezamelijk ruim 500 bewoners. Terminaal zieken, wachtend op verpleeghuisopname en ernstig cognitief gestoorden (MIS<2) worden geexcludeerd.
*. Interventie:*
Ketenzorg is op procesniveau geoperationaliseerd in drie sequentiele stappen: Ten eerste worden de gezondheidsstatus en zorgbehoefte van de bewoners getaxeerd door een verpleegkundige met het multidimensionele Resident Assessment Instrument (RAI). Het RAI wordt gecomputeriseerd afgenomen hetgeen is directe en gevalideerde probleemidentificatie mogelijk maakt en ziekte/disability management stuurt. De bevindingen worden besproken in een multidisciplinair overleg (MDO) waarbij de verpleegkundige, de huisarts, een verpleeghuisarts en psycholoog betrokken zijn. Het MDO maakt geindividualiseerde zorgplannen gericht op behandelbare of modificeerbare aandoeningen en risicofactoren.
*. Uitkomstmaten:*
primair: Quality of Care (Zimmerman criteria); Quality Adjusted Life Years (Euroqol); Functionele gezondheid (COOP-WONCA & SF12).
secundair: (I)ADL beperkingen(GARS); Zorgsatisfactie van bewoners (verkorte QUOTE); Stemming (PRiME-MD); Aantal opnamedagen in een ziekenhuis en tijd tot verpleeghuis, en sterfte.
*. Power-/data-analyse:*
We streven naar de inclusie van 166 bewoners hetgeen is berekend op detecteerbare verschillen met een effect size van 0.5. in de primaire maten (beta =0.2 & alfa=0.05 & anticiperend op intracluster inflatie 10% & 15% uitval).
*. Economische evaluatie:*
De kosten worden gemeten vanuit maatschappelijk perspectief. Zowel directe (bv zorgconsumptie) als indirecte kosten (bv zorgtijd door verzorgingshuispersoneel) worden gemeten.
*. Tijdplanning:*
totaal 24 maanden, 12 maanden inclusie, 6 maanden follow-up, 6 maanden analyses en rapportage.

**Key words**:
Homes for the elderly, integrated care, RAI, quality of life, functional health, prevention, nursing home admission

**Problem definition (50 lines)**:
*1. Describe the healthcare problem that underlies this proposal:*
Persons in the homes of the elderly suffer greatly from (multiple) chronic diseases and associated disablement (von Strauss 2003). In the usual care general practitioners are responsible but often unable to handle the often complex medical problems (De Burlet 1992, Challis 2003). GPs do not regard themselves to be suited for systematic management and long-term monitoring for chronic diseases and disabilities associated with frail health (Iliffe 2003). To increase the quality of care for disabled persons in homes for the elderly innovation is needed.

*2. What is the disease / condition, subject of this proposal:*
Chronically disabled but not terminally ill elderly are subject of this proposal.

*3. What is the (sub-) group of patients, targeted in this proposal:*
The patients in this proposal are residents in homes for the elderly. Homes for the elderly are predominantly populated with very old dependent persons, who suffer from (multiple) chronically disabling disorders. Over the last decades, residents have become older and more disabled and show more and more resemblance with nursing homes patients (Arcares 2004, NIVEL 2003).

*4. If applicable in relation to the target group, indicate how variations in sex, age or cultural background are taken into account:*
The persons in homes for the elderly in West-Friesland (as in the Netherlands as a whole) are predominantly white. Differences of effects for sex and age strata will be taken into account.

*5. Describe the usual care in the Netherlands for the (sub-) group of patients involved:*
GPs are responsible for the health care of elderly residents in homes for the elderly. Nevertheless many health problems go unnoticed for the GPs. The number of contacts with a GP depends largely on the preparedness of GPs to visit the homes. A system of multidisciplinary consultations is mostly absent. Most of the residents suffer from (multiple) chronic disorders.

*6. Who are involved in the usual care, and what is their participation in this proposal:*
GPs are responsible for the (usual) care. Occasionally, home care nurses, and nursing home physicians, or a nursing home psychologist may be consulted. Members of these groups participate in the current project. With the design of this study we anticipate on structural implementation in current health practice in West-Friesland. Representatives of patients, local GPs, health care organisation 'De Omring' which includes both homes for the elderly, nursing homes and home care, and the largest local health insurer (Univé) participate in the steering committee.

7. *Describe your motivation for the chosen innovation and the knowledge gap concerning the effectiveness of the innovation:*
Transmural Integrated Care (TIC) is strongly recommended to improve the health and quality of live of the chronically ill (NCCZ 1994, IGZ 2003, VWS 2004). However, no studies were performed yet to evaluate the cost-effectivenes of TIC in homes for the elderly. At this moment, the 20 homes for the elderly in West-Friesland offer a unique opportunity to evaluate TIC in a naturalistic quasi-experimental design. Half the homes belong to care organisation the ´Omring´ that comprises also nursing homes and home care. The 'Omring' has decided to set up TIC (ketenzorg) within its organisation and in close cooperation with other health professionals/ organisations to improve the quality of care. Therefore, agreements were made with the GPs, the mental health centre, the largest local health insurer (Univé) and the 'AWBZ zorgloket'. The other half of the homes belong to the 'Wilgaerde group', who offers residential homes only. They decided to stick to usual care. Therefore, all conditions are met for an optimal evaluation of TIC in residential homes in West-Friesland.
The TIC intervention in this proposal is tailored to the needs of patients and consists of several innovative elements: (1) TIC with an expanded multidimensional health perspective (that corresponds to the International Classifications of Functional Disabilities), (2) structural and computerised multidimensional geriatric screening in residential homes by trained staff, (3) patient tailored care plans made in multidisciplinary consultations including nurse staff, GPs, Nursing Home physicians, and psychologists, (4) the frailest residents with complex needs are offered a multidisciplinary consultation by a nursing home physician and psychologist. This broad and integrated approach in residential homes has not been evaluated yet.

**Relevance (50 lines)**
A. How will the results of the proposed study contribute to the resolution of this health care problem?
Transmural Integrated Care (TIC) is strongly recommended for chronic patients (NCCZ 1994, IGZ 2003). TIC is a disease driven and patient oriented approach, which contrasts with the current service driven system in which. Beneficial effects of TIC were reported among stroke patients (equal outcomes but fewer costs) and among diabetes mellitus type 2 patients (better outcomes)(Edisse 2002, Van Exel 2003, CvZ 2005). Very little is known about the cost-effectiveness of TIC in this setting.
Chronic disablement associated with aging is the main cost driver for national health care costs. Care facilities for elderly make up about one fifth of the costs of the Dutch health care system and are the second largest cost category after hospital care (Polder 2002). Within the care facilities for the elderly, residential homes are the largest cost category and take up about 40% of the budget(Polder 2002).

B. Are there any studies underway similar to the present study proposal or related to the healthcare problem of the present proposal?
Although TIC is strongly recommended to improve the quality of care of chronic patients, cost-effectiveness studies have not been performed in homes for the elderly. To our knowledge this will be the first study to evaluate the cost-effectiveness of TIC on chronic disabled persons in elederly homes.

C. Are there any recent reported by national advisory boards on the subject of your proposal?
Already in 1995 the National Health Council (Nationale Raad voor de Gezondheidszorg) stressed the importance of improving the quality of care for chronic patients by a shared disease management plan of the health professionals who are involved, with clearly defined medical responsibilities, and the development of shared management protocols (RVG 1995). In a recent report, published in 2003, on the state of health care by the Inspectie van de Gezondheidszorg (IGZ) alarming conclusions were drawn after rigorous investigations on the state of TIC (ketenzorg) in the Netherlands: Patients with chronic disorders are still at risk because of the lack of shared management by health professionals and health institutes, and unclear demarcation of medical responsibilities. There are insufficient guarantees for continuity of care between health professionals. The report concludes that without central control, integrated care will not have a (structural) future in our health care system. The minister of Health confirmed these alarming conclusions (VWS 2004).

D. What is the incidence / prevalence of the targeted population?
A chronic disorder is defined as all diseases that are not curable within six months or for which there is no prospect of full recovery (NCCZ 1994). It is estimated that 10-20% of the population of the Netherlands already suffers from a chronic disorder. In our aging population the number of persons with a chronic disease increases, between 1994-2015 with 25-60% (VTV 1997).
In the Netherlands there are about 110.000 residents in homes for the elderly (Arcares 2004). 71% of the residents need professional care such as assistance with activities of daily living, nursing care (medication, wound care), domestic help or mobility. Per 100 persons of 75 years or older 12 live in a home for the elderly and 6 in a nursing home (NIVEL 2004).
Incidence figures on elderly homes admission are not available but the generally long waiting lists (e.g. 350 days) suggest that the capacity does not match the demand (Nivel 2004, Arcares 2004).

E. Estimate the potential effects on health from the intervention that will be evaluated in this proposal compared to usual care.
Promising results were reported on TIC for stroke patients and for diabetes patients (Edisse 2000, Van Exel 2003, CvZ 2005). Intervention effects of TIC among disabled persons in homes for the elderly have not been studied yet. There are however, two meta-analyses on effects of home visits by nurses to community dwelling (frail) elderly (Stuck 2002, Elkan 2000). In a way such home visits can be regarded as TIC between nurses and GPs. Both meta-analyses concluded beneficial effects of preventive home visits on nursing home admission, and disability. The effects were stronger among frail elderly, and in studies that used a multidimensional assessment instrument (Stuck 2002). See also the systematic review in the strategy section.

F. Estimate the potential effects on costs from the intervention that will be evaluated in this proposal compared to usual care
Studies of comparable interventions and associated costs in residential homes are absent.
Nevertheless, we reanalysed two meta-analyses of Stuck 2002 and Elkan 2000 on preventive effects of home visits to community dwelling elderly and selected only studies that focused on frail elderly (12

out of 27 trials). Six studies reported overall direct health care costs and all interventions led to cost reductions (mean -15.2%; range -25 ; -4%).

**Kennisoverdracht (max 50 regels)**
** Formulate a clear aim for knowledge transfer and or implementation of the results of your proposal*
- If benefits of the interventions can be demonstrated, the aim for knowledge transfer is that half of the practicing GPs and management of homes for the elderly in the Netherlands take notice of results of this study. This will be accomplished by (lay, professional and scientific) publications, contributions to national symposia and education and training of GPs and GP trainees (at our department).
-For implementation in other parts of the Netherlands the current model can be copied without major alterations. Much depends of course of the spirit entrepreneurship within regions and cooperative attitudes of the organisations and persons involved.

* Who / What organisations should take notice of the study results?
-ZONmw
-Ministry of Health, Welfare and Sports
-Dutch College of Health Insurance (CvZ)
-Health insurers
-Dutch College of GPs (NHG)
-Dutch labour union of GPs (LHV and DHV-en)
-Arcares, national organisation for elderly and nursing homes
-NIZW
-Consensus and Guideline committees
-GPs
-Patient and consumer organisations
-Lay elderly and their family caregivers

* Who / What organisations should integrate the results in their activities?
- GPs
- Homes for the elderly
- Nursing homes
- Home Care Organisations

* How are these persons /organisations involved in the project?
GPs, Homes for the elderly, Nursing homes, Home Care Organisations participate actively in the current project. They employ the new transmural and multidisciplinary way of working instigated by this project. Representatives of patients, local GPs, care organisation De Omring (which include homes for the elderly, nursing homes and home care) participate in the steering committee.

**Objectives (max 25 lines)**
Persons in the homes of the elderly suffer greatly from (multiple) chronic diseases and associated disablement. General practitioners are responsible but often unable to handle the often complex medical problems especially those of the frailest residents. Therefore, Transmural Integrated Care (TIC) is strongly recommended for these persons. TIC (ketenzorg) is defined as a disease specific coherent package of care services delivered by various care providers. The perspective in this study, however, is expanded to biopsychosocial variables of disablement.
There are numerous regional initiatives in the Netherlands to create TIC for chronically ill. Promising results were reported on TIC for stroke patients and for diabetes patients. As yet little is known about the quality and effectiveness of TIC (ketenzorg) in homes for the elderly.
TIC is defined as a disease specific coherent package of care services delivered by various care providers. This may improve the health and quality of live of the residents and may be cost-effective.

Objectives:
1) To determine the effects and cost-effectiveness of integrated care for residents in homes for the elderly compared to usual care.
2) To identify residents who benefit most from the intervention.

**Strategy max 600 lines**
CLINICAL STUDY
* PRELIMINARY STUDIES BY APPLICANTS ON THE SUBJECT OF THIS PROPOSAL:
1. RAI assessment in residential home by staff members and multidisciplinary meeting:
Recently a pilot study was executed in one home for the elderly (St Martinus, Hoorn) among 53 residents. A staff member was trained to assess the Resident Assessment Instrument (RAI). All residents were assessed with the RAI under supervision of a nursing home physician. The detected health and care problems of the first four residents were discussed in a multidisciplinary meeting with their GP in a multidisciplinary meeting including the nurse and nursing home physician. 13 health problems emerged that were unknown to the GP such as severe depression, aphasia, severe hearing impairment, severe weight loss. The GP was very pleased with this new information and the advice on a management plan. Multidisciplinary meeting with GPs of the other residents are planned.

2. PIKO, an ongoing sister project among community dwelling frail elderly:
Our group currently executes a randomised trial to evaluate integrated (preventive) care among community dwelling frail elderly in West-Friesland. This project was started in July 2002 and involved close cooperation between the GPs and home care nurses. The functional health of all elderly of 75+ listed in 34 general practices was screened. Frail patients (worst quartile on COOP WONCA charts) were randomised to usual or integrated care. Nurses, trained to assess health status and care needs with the Resident Assessment Instrument (RAI), visited the patients of the integrated care group. Computerisation of the RAI enabled the immediate identification of care problems. Nurses determined the care priorities together wit the patient and design and execute protocolised care-plans. The nurses visit the patients at least five times during a year in order to execute and monitor the care-plan. The recruitment was recently closed and 683 patients were randomised.
In the meanwhile a lot of experience is gained with the RAI, making care plans and defining management protocols. Our main partner in this project is ´De Omring´, a health care organisation who trains and provides the home care nurses. In the current proposal 'De Omring' is involved again. ´De Omring´ comprises not only home care but the homes for the elderly and the nursing homes as well. Preliminary data showed an important reduction in the number of days spent in hospitals.

* DESIGN:
Cluster-randomised two group controlled clinical trial with 6 months follow up.

* RANDOMISATION

The randomisation is carried out per house and stratified on percentage of psychogeriatric residents. The care services and type of disability in houses with a high percentage of psychogeriatric patients differ between houses with few and houses with mangy residents needing psychogeriatric care. We assumed that these differences can substantially influence the outcomes measured. Following earlier reports on underservice of complex residents, we hypothesized that higher resident complexity will show more benefits from our care intervention. So, the houses were fist ranked on percentage of psychogeriatric patients. The two houses with the highest percentage of psychogeriatric patients were than matched, on to the two houses with the lowest percentage of psychogeriatric patients.
Next, we check the risk of imbalance in numbers. If the difference in number of intervention and control residents is >10% (50 or more) the randomisation must be repeated until the imbalance is 10% or less.
Houses are all ordered on percentage psychogeriatric patients and numbered from rank one to rank 10. In this way matched houses are ranked after each other, one having an even and the other an uneven number. Randomisation is carried out using Pocock's first column in his random numbers table (Pocock 1983). If the table's first number is even, the even number of first matched house is assigned the intervention. If the next table number is uneven, the uneven number of the next matched couple is assigned the intervention. And so on until all matched couples are assigned.

* SETTING
Ten homes for the elderly in West-Friesland offer a unique opportunity to evaluate TIC in an experimental design.
Care organisation the 'Omring' in West-Friesland has decided to set up TIC (ketenzorg) within its organisation and in close cooperation with other health professionals/ organisations to improve the quality of care. Agreements were reached with the GPs, the mental health centre, the largest local health insurer (Univé) and CIZ, the AWBZ care indication office. Financial issues were resolved i.e.

the GPs who spend time on multidisciplinary meetings are compensated. The time of home care nurses who assess residents with the RAI as well as the consultations by the nursing home physicians can be declared at the ‘AWBZ-Zorgloket‘.
In order to enable the evaluation of TIC, the ‘Omring; has agreed to implement TIC in half their homes and keep usual care in the other half until the evaluation measurements are done.

* STUDY POPULATION:
All residents from 10 homes for the elderly in the region West-Friesland are eligible. Exclusion criteria are terminally ill or end stage disease, severely cognitively impaired (Memory Impairment Screen<2), and persons who are on the waiting list for a nursing home. Terminally ill is defined as severely diseased persons who have no therapeutic perspective and are expected to die within 6 months according to the GP or residential staff.
The TIC intervention is executed in five ‘Omring houses’ which comprise about 250 residents.
The usual care homes will be five ‘Omring houses’ also in West-Friesland, which also comprise about 250 residents. We expect that about 300 persons are eligible and willing to participate which exceeds the 166 needed participants.

* INTERVENTION:
TIC (ketenzorg) is a disease specific coherent package of care services delivered by various care providers. We defined Transmural Integrated Care (TIC) as providing continuity of care, being patient oriented, generating multidimensional health data on patients’ health, is executed by appropriately trained professionals who design a shared disease management plan and is preferably ICT supported.
Transmural Integrated Care (TIC) is operationalised on several levels.
-In the homes:
We operationalised TIC in the process of care in three sequential steps:
1. Firstly, an in home-visit of staff (nurse) who identifies care needs of all residents with a computerised multidimensional health assessment. For this purpose, the Resident Assessment Instrument (RAI) will be used (www.rai.org). The RAI was originally designed, as a minimum data set to assess the health needs of nursing home residents. For homes for the elderly an adapted version is available. The RAI provides a comprehensive overview of the patient's physical, psychological, behavioural, social status. Moreover it indicates a global level of care need which distinguishes persons who do not need care, from those who need personal care, home care, extra home care or nursing home care.
The computerised RAI enables an easy and direct overview of problems spotted in 18 different areas. The problem areas guide the design of an optimal individualised care plan. In a multidisciplinary team, all disciplines involved in residential care, will participate in regular meetings in order to evaluate the RAI findings and design and monitor the (tailor made) care-plan. The care plan is focused on modifiable disabilities and risk factors of the resident.
2. Secondly, the assessment outcomes are discussed in multidisciplinary meetings (MM) in the homes with the GP, nursing home physician, nurse and psychologist. In the MC an individualised care plan is made to treat modifiable disabilities and risk factors. Treatment plans will follow protocols whenever possible. The ACOVE recommendations will be used as guidelines for treatment plans (Saliba 2005).
3. Thirdly, a multidisciplinary consultation (nursing-home physician and psychologist) is offered to the frailest residents with complex health care problems. They are identified by the level of care need indicator of the RAI as needing nursing home care.

In addition, the computerised RAI also provides process-supporting information technology as well as indicators about the functioning and implementation of the care plans. A limitation of TIC is the often single-disease oriented perspective. Therefore, in this project among elderly with multiple morbidity, we chose an expanded multidimensional or biopsychosocial perspective which corresponds to the International Classification of Functioning, Disability and Health (ICF) (WHO 2001).

-In the contextual setting:
TIC in homes for the elderly in West-Friesland further meets important conditions for complex organisational interventions. All parties involved participate in this project (i.e. GPs, homes for the elderly and their nursing staff, nursing homes physicians, and home care). Also, potential financial barriers were discussed with the parties and were beforehand resolved i.e. GPs are compensated for their time in the multidisciplinary consultations by the main local health insurer (Unive), and budget was reserved by the local AWBZ zorgloket for nurse assessors and Nursing Home physicians.

With the design of this study we anticipate on structural implementation in current health practice in West-Friesland. Representatives of patients, local GPs, health care organisation ‘De Omring’ which includes both homes for the elderly, nursing homes and home care, and the largest local health insurer (Univé) participate in the steering committee.

* USUAL CARE:
The 5 homes for the elderly will continue to offer usual care to their residents until the outcome measurements are completed. The GP is primary responsible for the health care of these residents. In some houses special care wards are installed for very frail or demented persons. A system of multidisciplinary consultations is absent.

* INNOVATIONS
-This is the first evaluation of TIC in homes for the elderly
-The intervention is performed on both organisational and process of care level.
-Contrary to current single-disease oriented TIC interventions, we use an expanded multidimensional or biopsychosocial perspective which corresponds to the International Classification of Functioning, Disability and Health (ICF) (WHO 2001). We consider this more appropriate for frail elderly with multiple morbidity.
-The computerised RAI provides process-supporting information technology as well as indicators about the functioning and implementation of the care plans.

* OUTCOMES AND MEASUREMENTS (table 1)
Primary:
a. Quality of Care (Zimmerman criteria 1995)
b. Quality Adjusted Life Years (QALY’s) by EuroQol (Euroqol group 1990).
c. Functional health by COOP-WONCA & SF12 (Van Weel 1995, McHorney 1985)
Secondary
d. Disability by GARS ((instrumental) activities of daily living) (Kempen 1993)
e. Care satisfaction of residents by brief QUOTE (Sixma 2000)
e. Mood, Depressive and Anxiety disorders by PRiME-MD (Spitzer 2000)
f. Days spend in Hospital
g. Time to nursing home placement
h. Time to mortality
Process outcomes:
i. Adherence of physicians, nurse staff to TIC protocol (e.g. performance of RAI screening, MC, and availability care plan)
j. Adherence of patients to specific TIC recommendations

Potential effect modifiers are measured and are sought in: demographics, cognitive status (MIS), chronic diseases, medication use, house characteristics, GP characteristics and attitude on elderly care.

* SAMPLE SIZE CALCULATION AND FEASIBILITY OF RECRUITMENT:
Sample size calculations were based on the expected effects of the intervention on the main outcome measures, quality of life utility the functional health score, and on resource use of the main cost drivers (hospital and nursing home stay). Effect estimates are based on our update of the meta-analyses/ reviews of Stuck (2002) Elkan (2000) and Johri (2003) of integrated preventive interventions among (frail) elderly. A limitation of these studies is that they concerned frail elderly living at home instead of in homes for the elderly.
In the following sample size calculations we used an alpha of 0.05, power of 80% and inflation of 10% because of anticipated intracluster correlation in the elderly homes. Regarding health related quality of live, effect sizes ranged from 0.5 to 3.8 in our meta-analysis. If we anticipate detecting a fair benefit, i.e. effect size=0.5, a minimum of 64 persons is needed in each group (Cohen 1977). For functional health and disability we anticipate on a comparable effect-size and consequently sample size. If we assume a dropout rate of 15% during the 6 months follow-up we need to include 100/85 x 64 x 2 x 110% = 166 persons.
Although it concerns a secondary outcome measure we also estimated the required sample size to detect differences in resource use. For the number of hospital days, an annual difference of 79 days (SD 78) per 100 persons was found in our meta-analysis. Therefore, 108 persons per group are needed in order to reach significance for a difference of 79 hospital days (Statpower software). If we

assume a dropout rate of 20% during the 6 months follow-up we would need to include 100/80 x 108 x 2x 110% =297 persons. Regarding nursing home days an annual difference of 2.7days (SD 43) per 100 persons was found in our meta-analysis. Therefore, more than 4000 persons per group would be needed in order to reach significance for this difference (Statpower software). The latter numbers are beyond reach in our study.
In conclusion, we aim to include 166 persons, which seems feasible regarding the potential participation of 10 homes for the elderly that comprise over 500 persons. In our pilot, all 53 residents of a home for the elderly participated in RAI assessment.

* DATA ANALYSIS
Data will be primarily analysed according to the Intention to treat principle, i.e. including all participants with valid data, regardless of whether they received or did not receive the intervention. Subsequently, the results of the intention to treat analysis will be compared with the results of the 'on treatment' analysis, to assess whether protocol violations have caused bias. Participants (or houses) with documented deviations from the protocol (i.e. participants who did not receive the entire intervention or participants in either the intervention or the control group with incomplete follow-up data) will be excluded from the on treatment analysis. Comparability between the intervention and control groups will be assessed at baseline to check differences.
Outcomes at 6 months will be compared between intervention and control groups by both univariate and multivariate techniques. We will use the multivariate technique to adjust for possible differences in baseline scores and background variables between the intervention and control groups. Dropout and loss to follow up will be described.

* PROCESS EVALUATION
The process evaluation involves assessing the extent to which the intervention programme is performed according to protocols, the nature of the recommendations made to the participants, the participants' compliance with these recommendations and the opinions of participants, physicians, and therapists about the intervention programme and recommendations. Data on these topics are collected using the following methods; structured registration forms for the disease management parts of the TIC; self administered evaluations forms filled in by the participants after the TIC intervention; interviews with the participating nurses, GPs, nursing home physicians at the end of the intervention period.

ECONOMIC EVALUATION
* *General considerations:*
Cost data are collected by patient interview at baseline, and at 3 and 6 months from a societal perspective. In case patients are not competent or not able anymore to be interviewed, proxies will be sought, preferably close family members. The following costs will be considered: 1) direct healthcare costs, such as costs of consultations of the general practitioner, nursing home physician, medical specialist, hospitalisations, and medical department of the nursing home, and use of medication and medical aids (hulpmiddelen) 2) direct non-healthcare costs (time and travel costs of the patient and his family/mantelzorgers) and 3) indirect costs, such as costs of informal care. Medication data are retrieved from the centralised pharmacy files in West-Friesland. If available, Dutch guideline prices are used to value resource use (Oostenbrink 2000 & 2002). Otherwise, tariffs are used. Medication costs are valued using prices of the Royal Dutch Society for Pharmacy (Z-index 2004). Contacts with GPs and referrals will be checked as well in GPs patient information files.

* *Cost analysis:*
To compare costs between the two groups, confidence intervals for the differences in mean costs are calculated using bias-corrected and accelerated bootstrapping with 2000 replications (Efron 1993). For the cost-effectiveness analysis the difference in total costs between the intervention and usual care group are compared with the difference over 6 months in improvement of functional health and disability. In addition, a cost-utility analysis will be done to assess the incremental costs per QALY. Uncertainty around the cost-effectiveness and cost-utility ratios is calculated using the bias-corrected percentile method (5000 replications) and presented in a cost-effectiveness plane (Chaudhary 1996).

* *Patient outcome analysis:*
Quality Adjusted Life Years (QALY s) are calculated by multiplying the utility based on EuroQol scores (Dolan 1997) with the amount of time a patient spent in this particular health state. Transitions between health states are linearly interpolated.

TIME SCHEDULE
Total 24 months:
12 months baseline measurements of 166 residents (=14 a months), 6 months follow up, 6 months analyses and reports.

**SYSTEMATIC REVIEW**
** Introduction*
We first searched Pubmed for controlled trials on transmural integrated care in homes for the elderly. Basically, our search key combined terms for controlled trials with homes for the elderly and with transmural or integrated care. However, we did not find any controlled trials on this subject let alone economic evaluations on this subject. Nevertheless, we did find controlled trials on integrated care for home-dwelling elderly. We decided to review the evidence of these trails and focus on vulnerable or frail elderly only.

** Search*
On this subject two meta-analyses and one systematic reviews were published previously. Elkan et al. searched up to 1998, Stuck et al. and Johri et al. searched up to 2000.
Regarding economic evaluations on this subject, to our knowledge, no review was performed yet. Therefore we decided to perform two searches. The first was an update search on the clinical effects on integrated care among frail elderly at home starting in 1998. The second search on economic evaluations of this subject was performed without time constraints. No language limitations were used. The supplement (search PIKOV HvH) shows our detailed search keys for digital databases. Basically we combined keywords for RCT, nurse visits, geriatric assessment and aged. For the economic evaluation the same strategy was used while adding economic terms. Two reviewers independently judged the appropriateness of the studies.

** Population:*
-Frail community or general practice patients (e.g. bad self-reported health, needing home care, discharged from hospital)
-Patients were 65+ years or older or had a mean age of 65 or higher.

** Intervention:*
-Evaluating a transmural home visiting programme (e.g. close cooperation between district nurses and GPs)
-Home visitors had to undertake geriatric assessment and provide surveillance, support, health promotion and the prevention of ill health.
-The intervention had to involve the pursuit of a wide range of preventive outcomes rather than a single goal (e.g. not only prevention of falls or high blood pressure).

**  Comparison/control:*
Comparisons were made on controls receiving usual care.

**  Outcome:*
-Outcomes on either: quality of Life, nursing home admission, hospital admission, and mortality
-As additional criterion only for the economic evaluation a study should report at least volume or cost data on nursing home and hospital stay. They can be considered the main cost drivers.

** Methodological filters:*
We searched in Pubmed, Psychlit, CINAHL, the Cochrane trial register and the CRD database (DARE, NHS EED, HTA). We checked the references from the two meta-analyses and papers on the subject already known to us.

** Selection procedure, validity assessment*
We included only empirical studies describing (randomised) clinical trials. We excluded patient populations selected on a specific disease (e.g. asthma, arthritis, depression, dementia, heart failure) and studies that reported only very specific outcomes (e.g. hypertension, cholesterol, falls, satisfaction). The data extraction was double checked by two reviewers Methodological quality of the studies was checked according to Reish' criteria (range 0-1). Meta-view 4.2 was used to calculate pooled odds ratio for binary outcomes and standardised mean differences for the continuous

outcomes. Excel was used to calculate annualised differences in the number of hospital and nursing home days.

** Results*
(Primary outcome parameter / Secondary outcome parameters / Economic evaluation)
The search resulted in 140 potentially relevant reports, 41 were retrieved for detailed study, 12 were eligible and contained relevant clinical outcomes, 9 of these also reported cost data and/or information on resource utilisation concerning at least one of the main cost drivers (number of hospital days and/or nursing home days) (Table 2).

*-Clinical effects*
Nursing home admission: Seven trials on frail elderly, sufficiently homogeneous to pool, reported a mean annual reduction in admission of 4.7% among intervention patients compared to usual patients with a risk reduction of 0.66 (95% CI 0.46-0.95).
Hospital admission: Four trials on frail elderly, sufficiently homogeneous to pool, reported a mean annual (non-significant) reduction among intervention patients compared to usual care of 6.7% with a risk reduction of 0.89 (0.58-1.37 NS).
Quality of live: Three trials also reported on the quality of live of frail participants. The measures they used were, however, all different and to heterogeneous to pool. The standardised mean differences ranged from moderate benefit (effect size=0.5) to substantial benefit (effect size=1.3).

*-Economic effects*
In addition, we were able to extract cost information on at least one of the main cost drivers (hospital and nursing home stay) from 9 out of the 12 included studies.
Overall costs: Six studies reported overall direct health care costs. All reported cost reductions (median -15.2%; range -25 ; -4%).
Cost drivers: Nine studies reported on the number of hospital days. The median annual reduction in hospital days was 67 days per 100 frail persons per year (range 0-184). Only two studies reported on the number of nursing homes days. One found a beneficial annual difference of 6.5 days, the other found a very small annual difference in favour of the usual care of 1.2 day. The median annual (non-significant) reduction in nursing home days was therefore 2.7 days per 100 frail persons.

**Expertise, prior activities, products (max 75 lines)**
EMGO INSTITUTE: The Institute for Research in Extramural Medicine (EMGO) is one of five research institutes of the VU University Medical Center Amsterdam. Our activities predominantly deal with research in primary and transmural care, and public health, focusing on chronic diseases and ageing. It concerns multidisciplinary applied research with a strong emphasis on health outcomes relevant to individuals in the extramural setting. The EMGO Institute collaborates intensively with many national and international partners and is one of the founding participants of the Netherlands School of Primary Care Research (CaRe). Our dual aim is to excel on criteria for scientific quality as well as on criteria for societal relevance. The EMGO-Institute has a quality policy in which a special committee issues standard operating procedures (SOP) on all aspects of research. The committee further monitors and audits SOPs, which reflect good clinical practice. The EMGO Institute was recently reviewed as part of the assessment system for Dutch universities and achieved the highest status (excellence).
PROGRAMMES: The research projects are grouped in four consistent programmes. The current proposal is embedded in the programme ‘Care and Prevention’(C&P). C&P involves multidisciplinary collaboration between the disciplines of general practice, nursing home medicine, medical psychology and psychiatry. C&P concerns both large longitudinal community studies as intervention studies in residential homes, nursing homes and among family caregivers. Research projects in this programme are clustered in four domains which, in turn, are inter-related: (1) quality of care and prevention, (2) end-of-life care, (3) functional autonomy and the course of chronic disease, and (4) life-style and health.
RESEARCH GROUP: The research group has an excellent record of population studies in different areas. The project leader Dr. G. Nijpels is leader of the Hoorn study a longitudinal cohort study on diabetes with extensive publications in top-journals. Diabetic patients in the Hoorn study were the first to profit from a transmural integrated care model with close cooperation of, among others, GPs, home care nurses and specialists. He is also general practitioner and a central figure in the network of care organisations in West-Friesland. He currently supervises a ‘CBO doorbraak’ project on diabetes care in Amsterdam in order to set a transmural integrated care model with close cooperation of the VUmc, Home care organisation, and GPs.

The other project leader, Dr. H. van Hout, has published extensively on dementia and depression in primary care. He is involved in the Longitudinal Aging Study Amsterdam (LASA) as well. LASA is an internationally highly respected study, which published extensively on frailty and disability, in relation to care-needs and care-use of elderly. Dr. H. van Hout participated in a European study of the effects of health promotion for elderly persons (Prof. Dr. Stuck, Bern). He is also member of INTERDEM, a European expert group on psychosocial interventions for dementia patients and their caregivers. Professor Stalman, head of the department of general practice, chairs the VUmc committee 'Transmuraal' that aims to achieve transmural cooperation within the VUmc as well as with the local GPs. He also chairs of the national CBO guideline committee on cardiac risk management that aims to make a comprehensive (transmural) guideline in which all medical disciplines are represented. Professor Ribbe, head of the department of nursing home medicine, has an excellent record of intervention studies among frail elderly in nursing homes. His department introduced the Resident Assessment Instrument (RAI) in the Netherlands, which is studied in an international co-operation. Project member, Dr Harm van Marwijk, is an expert on old age depression and first author on NHG and CBO guidelines on depression. He is project leader of several evaluation of transmural intervention projects on mental health problems in primary care. Dr Martine de Bruijne and Dr Maurits van Tulder are senior HTA researchers and have extensive experience on economic evaluations. PRIOR ACTIVITIES: The department of Nursing Home Medicine introduced and developed the Resident Assessment Instrument (RAI) in the Netherlands. We are involved with the integration of the RAI in several care organisations. A sister project, 'Preventieve Interventie voor Kwetsbare Ouderen' (PIKO) runs currently among 600 frail community dwelling elderly (Directors Dr. G Nijpels & Dr. H. van Hout) It involves integrated multidisciplinary care by GPs and district nurses. Therefore we gathered extensive expertise on all aspects of complex intervention on integrated care i.e. computerised RAI assessment by nurses, communication between care partners, developing shared protocols, quality control.
CARE LINKS: The proposal fits in with a local care initiative in West-Friesland with participation of all residential homes and general practitioners. In addition nursing homes provide multidisciplinary consultations and the home care organisations 'De Omring' provides training of the nurses. Preparing this care initiative, all 20 homes for the elderly, the regional nursing homes, the regional home-care organisation (Omring Thuiszorg) and all 59 regional operating GPs agreed to participate with this project. Also the main healthy insurer agreed to compensate GPs for the time they spend on the multidisciplinary consultations. The local 'AWBZ zorgloket' agreed to compensate for time of district nurses and nursing home physicians in his project. Chances for implementation are therefore very good.

**References: (max. 75 lines)**


- Arcares Verpleeghuizen & verzorgingshuizen in cijfers. www.arcares.nl 2004
- Burlet de, HM. Ouderdom: altijd gebreken? Dissertatie EUR, Rotterdam 1992
- Challis D, Hughes J. Frail old people at the margins of care: some recent research findings. Br J Psychiatry. 2002;180:126-30.
- Challis D, Hughes J. Residential and nursing home care-issues of balance and quality of care. Int J Geriatr Psychiatry. 2003;18:201-4
- Chaudhary MA,.Stearns SC. Estimating confidence intervals for cost-effectiveness ratios: an example from a randomized trial. Statistics in Medicine 1996;15:1447-58.
- Cohen J, Statistical power analysis for the behavioural sciences. Academic press, New York/London 1977.
- CVZ, College voor Zorgverzekeringen, Evaluatie van ketenzorg bij diabetes in Nederland. Report In progress 2005.
- Dolan P. Modeling valuations for EuroQol health states. Med Care 1997;35:1095-108.
- EDISSE, Beroerte, beroering en borging in the keten, Resultaten van de EDISSE studie van drie regionale experimenten met stroke service, ZOMmw rapport Den Haag 2002.
- Efron B, Tibshirani RJ. An introduction to the bootstrap. New York London: Chapman & Hall, 1993.
- Elkan R, Kendrick D, Dewey M, Hewitt M, Robinson J, Blair M, Williams D, Brummell K. Effectiveness of home based support for older people: systematic review and meta-analysis. BMJ 2001; 323: 719-725
- Frijters D et al. Geïntegreerd gezondheids-informatiesysteem op basis van Resident Assessment Instrument. Tijdschr Gerontol Geriatr 2001; 32: 8-16
- IGZ, Inspectie voor de Gezondheidszorg, The State of Health Care: coordination of care (seamless) for chronically ill patients. Den Haag 2003 www.igz.nl.

- Iliffe S, Manthorpe J, Eden A. Sooner or later? Issues in the early diagnosis of dementia in general practice: a qualitative study. Fam Pract. 2003;20:376-81.
- Johri M, Beland F, Bergman H. International experiments in integrated care for the elderly: a synthesis of the evidence. Int J Geriatr Psychiatry. 2003;18:222-35.
- Kempen G, Doeglas D, Suurmeijer Th, Het meten van problemen met zelfredzaamheid op verzorgend en huishoudelijk gebied met de Groninger Activiteiten Restrictie Schaal (GARS), Northern Centre for Health Care Research, University of Groningen. 1993.
- Lagaay AM, van der Meij JC, Hijmans W. Validation of medical history taking as part of a population based survey in subjects aged 85 and over. BMJ. 1992;304:1091-2
- NCCZ, National Committee for the Chronically Ill, Chronische zieken aan zet: Tussen balans chronisch ziekenbeleid (1991-1994) Zoetermeer 1994.
- NIVEL Feiten en Cijfers, Chronisch zieken kort en bondig, www.nivel.nl Utrecht 2003
- NIVEL Feiten en Cijfers, Vraag en gebruik: Voor welke gezondheidsproblemen wordt verpleeghuis- en verzorgingshuis gebruikt?, www.nivel.nl Utrecht 2003
- NRV, Nationale Raad voor de Volksgezondheid. Transmurale somatische zorg. Advies van de Nationale Raad voor de Volksgezondheid en het College voor ziekenhuisvoorzieningen. Zoetermeer/Utrecht: NRV/CvZ, 1995. www.rvz.net
- NSII, Cardol M, Van Dijk L, De Jong JD, De Bakker DH, Westert GP, Tweede Nationale Studie naar Ziekten en Verrichtingen in de huisartspraktijk: Huisartsenzorg, wat doet de huisarts. NIVEL, Utrecht 2004.
- Oostenbrink JB, Koopmanschap MA, Rutten FF. Handbook for cost studies, methods and guidelines for economic evaluation in health care [Handl;eiding voor kostenonderzoek, methoden en richtlijnprijzen voor economische evaluaties in de gezondheidszorg]. Health Care Insurance Council [College voor Zorgverzekeringen] 2000.
- Oostenbrink JB, Koopmanschap MA, Rutten FF. Standardisation of costs: the Dutch Manual for Costing in economic evaluations. Pharmacoeconomics 2002;20:443-54.
- Polder JJ ; Achterberg PW Cost of illness in the Netherlands RIVM Rapport, Bilthoven 2004 www.rivm.nl / www.costofillness.nl
- Shepperd S, Iliffe S. Hospital at home versus in-patient hospital care. Cochrane Database Syst Rev. 2001;(3):CD000356.
- Sixma HJ, van Campen C, Kerssens JJ, Peters L. Quality of care from the perspective of elderly people: the QUOTE-elderly instrument. Age Ageing. 2000;29:173-8.
- EuroQol Group. Euroqol - a new facility for the measurement of health related quality of life. Health Policy 1990;16:199-208.
- Saliba D, Solomon D, Rubenstein L, Young R, Schnelle J, Roth C, Wenger N. Quality indicators for the management of medical conditions in nursing home residents. J Am Med Dir Assoc. 2005;6(3 Suppl):S36-48.
- Stuck AE, Egger M, Hammer A, Minder CE, Beck JC. Home visits to prevent nursing home admission and functional decline in elderly people. *JAMA* 2002; 287: 1022-1028
- Van Exel J, Koopmanschap MA, Van Wijngaarden JD, Scholte Op Reimer WJ. Costs of stroke and stroke services: Determinants of patient costs and a comparison of costs of regular care and care organised in stroke services. Cost Eff Resour Alloc. 2003;1:2.
- Van Weel C, Konig-Zahn C, Touw-Otten F, van Duijn N, Meyboom-de Jong B, Measuring functional health with the COOP/WONCA charts, a manual, Northern Centre for Health Care Research, University of Groningen. 1995
- Van der Zee KI, Sanderman R, Het meten van de algemene gezondheid met de RAND-36, een handleiding. Noordelijk Centrum voor Gezondheidsvraagstukken, Groningen 1990.
- von Strauss E, Aguero-Torres H, Kareholt I, Winblad B, Fratiglioni L. Women are more disabled in basic activities of daily living than men only in very advanced ages: a study on disability, morbidity, and mortality from the Kungsholmen Project. J Clin Epidemiol. 2003;56:669-77.
- VTV, Volksgezondheid Toekomst Verkenning 1997 VI Zorggebruik en Zorgbehoefte, Red: Post D, Stokx LJ, RIVM Bilthoven Elsevier/De Tijdstroom 1997.
- VWS, reactie van Hans Hogervorst op het rapport Staat van zorg 2003 van de Inspectie van de Gezondheidszorg. Den Haag 2004.
- WHO ICF World Health Organisation, International Classification of Functioning, Disability and Health. http://www3.who.int/icf/icftemplate.cfm
- Zimmerman, D, SL. Karon, G Arling, BR Clark, T Collins, and R Ross. Development and Testing of Nursing Home Quality Indicators. Health Care Financing Review 16.4 (1995): 107-127.
- Z-index. G-Standaard. The Hague, The Netherlands: Z-index, 2002.

**Figure 1.** Flow chart PIKOV: Preventive effects of Transmural Integrated Care (´ketenzorg´) on disabled persons within homes for the elderly, a controlled clinical trial

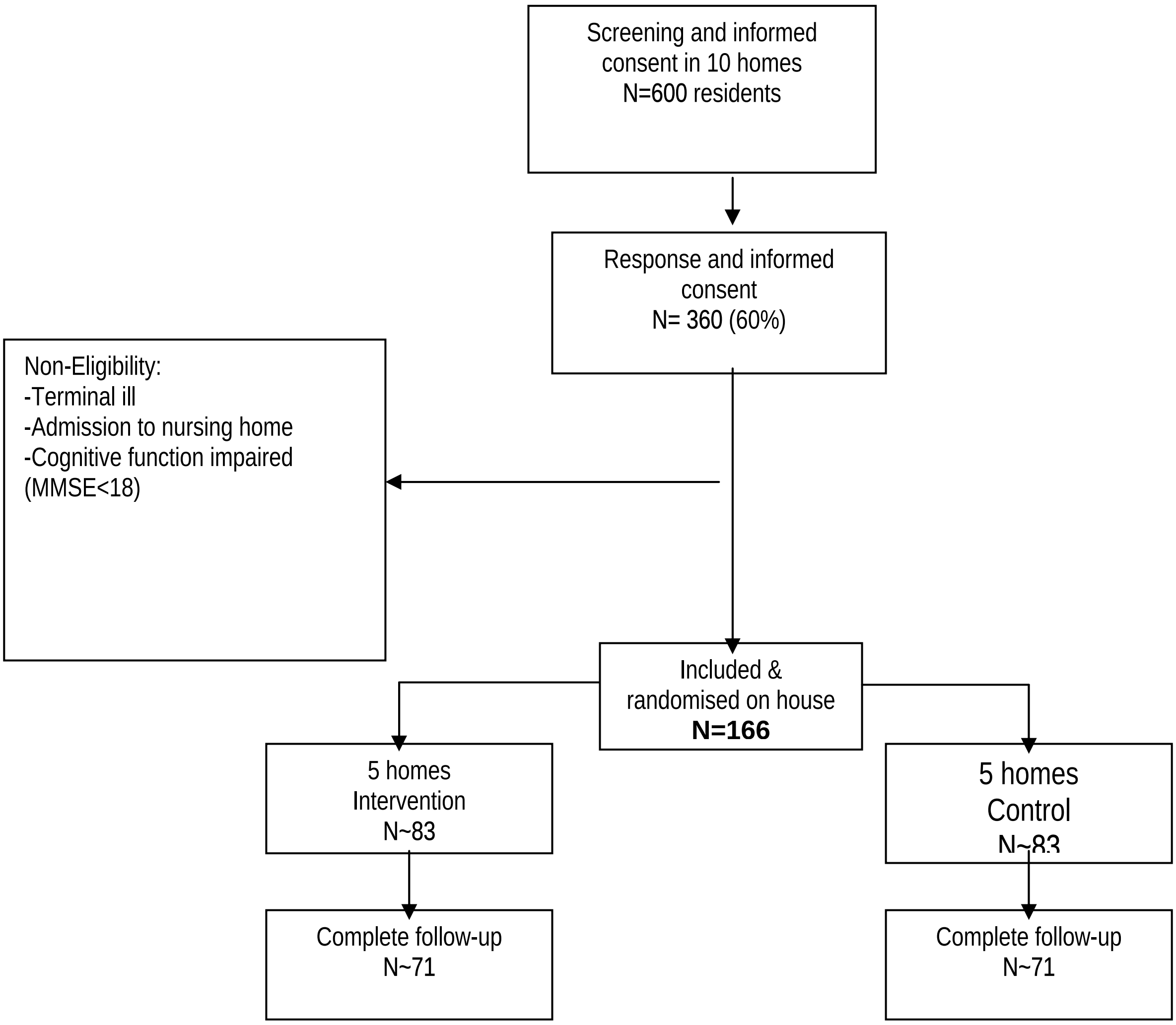

**Table 1. Measurements PIKOV:** April 2006
**Preventieve Interventies bij Kwetsbare Ouderen in Verzorgingshuizen**

| Variable | Instrument | *Items* | *Interview / Question / Registration Observation* | *Time per patient min.* | *Frequency* | |
|---|---|---|---|---|---|---|
| *Exclusion* | | | | | T0 | T1 |
| Terminal <6 months (wel in zzo?!) | Personel / GP | 1 | R / O | 0 | X | |
| *Primary outcome* | | | | | | |
| a. Quality of Care | RAI (Zimmerman criteria) | 29 | O / I | 5 | X | X |
| b. Quality Adjusted Life Years | *Euroqol +thermo* | 6 | I | 2 | X | X |
| c. Functional health | *COOP-WONCA & SF12* | 5-12 | I | 2 | X | X |
| *Secondary outcome* | | | | | | |
| d. Disability (ADL & IADL) | GARS | 18 | I | 5 | X | X |
| e. Patient care satisfaction | brief QUOTE on elderly home | 16 | I | 5 | | X |
| f. Mood disorders (zzo) | PRIME-MD | 8 | I | 5 | X | X |
| g. Hospital days | Zorgdagboek,<br>Hospital records | .. | I<br>R | ..<br>.. | X | X<br>X |
| h. Time to nursing home placement | House registration<br>HIS | Nvt | R<br>R | .. | | X<br>X |
| i. Time to mortality | House registration<br>HIS | Nvt | R<br>R | .. | | X<br>X |
| j. Direct & indirecte costs | Checklist Zorggebruik<br>Registratie apothekers<br>Zorg registratie Huis | 20<br>1<br>… | I<br>R<br>R | 5<br>.. | X | X<br>X<br>X |
| *Potential modification/ confounding* | | | | | | |
| Sociodemographics<br>Cognition<br>Medication use<br>Perceived self-efficacy<br>Loneliness<br>Characteristics Elderly home<br>Characteristics GP | Demographics<br>MIS<br>Interview / pharmacist<br>Mastery<br>De Jong-Gierveld<br>….<br>Demographics /attitude | 8<br>1<br><br>5<br>11<br>?<br>5 | I<br>I<br>I/R<br>I<br>I<br>R<br>Q | 2<br>4<br>5<br>2<br>3<br>0<br>0 | X<br>X<br>X<br>X<br>X<br>X<br>X | <br>X<br>X |
| *Additional measures (ook ZZO)* | | | | | | |
| -Objective care needs<br>-Subjective care needs (ZZO) | RAI*<br>CANE * | 336<br>24 | O<br>I | 0*<br>30* | X | X |
| *Total* | | | | 45 + 30 | | 38* |

*T0:* *baseline measurement*
*T1:* *follow-up at 6 months*

** Intervention group only*
*Time needed to complete the RAI, this will mainly be based on observations by personnel. Its burden for patients will therefore be limited.*

**Table 2. REVIEW**
Clinical & Economic effects of preventive home visits to frail elderly

| | CLINICAL OUTCOMES | | | | | | | | ECONOMIC OUTCOMES | | | |
|---|---|---|---|---|---|---|---|---|---|---|---|---|
| | **Sample size** *Ni / Nc* | **Nursing home admission** *ni/nc* | *OR* | **hospital admission** *ni/nc* | *OR* | **Mortality** | | **Quality of life** | | Total health care costs % %i - %c | % Annual Nursing Home days/ 100pat/ ni – nc | % Annual Hospital days/ 100pat ni - nc |
| Balaban 1988 | 103 / 95 | | | | | 31/20 | 1,61 | | | | | -12 |
| Bernabei 1998 | 99 / 100 | 10/15 | 0,64 | 36/51 | 0,55 | | | 3,8 | | -22,0 | | -154 |
| Cohen 2002 | 317 / 318 | | | | | 64/64 | 1,00 | 0,27a | | -4,4 | | -13 |
| Dunn 1990 | 102 / 102 | 8/7 | 1,16 | | | 15/25 | 0,53 | | | | | -121 |
| Hall 1992 | 81 / 86 | 6/17 | 0,32 | | | 21/34 | 0,54 | | | | 1,2 | |
| Hansen 1992 | 163 / 181 | 16/29 | 0,57 | 91/101 | 1,00 | 32/43 | 0,78 | | | | | 0 |
| Hebert 2001 | 250 / 253 | 5/5 | 1,01 | | | 12/18 | 0,66 | | | | | |
| Landi 2001 | 88 / 88 | | | 31/23 | 1,54 | 27/26 | 1,06 | | | -21,0 | -6,5 | -150 |
| Oktay 1990 | 285 / 287 | 37/45 | 0,80 | | | 27/28 | 0,97 | | | -23,0 | | -190 |
| Robichaud 2000 | 35 / 45 | 1/5 | 0,30 | 11/18 | 0,69 | 1/5 | 0,24 | -0,41 | | -7,3 | | -184 |
| Rubin 1992 | 91 / 87 | | | | | | | | | -24,8 | | -151 |
| Williams 1992 | 231 / 239 | | | | | | 0,74 | -0,21b | | | | -3 |
| **Total** | **1845 / 1881** | **83/122** | | **169/193** | | **260/303** | | | | | | |
| | | | | | | | | | **Mean** | **-17,1** | **-2,7** | **-97,8** |
| **pooled OR (95%CI)** | | **0.66 (0.47-0.93)** | | **0.89 (0.58-1.37)** | | **0.83 (0.65-1.06)** | | | | | | |
| | | | | | | | | | **Median** | **-21,5** | **-2,7** | **-135,2** |
| **Overall effect** | | | **p=0.02*** | | **p=0.6** | | **p=0.14** | | **SD** | **8,8** | **5,4** | **80,5** |

Ni / Nc = sample size of intervention group versus control group
OR (95%CI)=Odds Ratio with 95% Confidence Interval
a=eight domains on the SF36 were presented with median effect size 0,27 (range 0,0-1,6)
b=three domains were presented with median effect size -0,21(range-0,44-0,15)
*=significance <0.05
SMD=standardised mean difference (mean control minus mean intervention divided by pooled standard deviation)
